# Predictive value of decreased p27^Kip1^ protein expression for the recurrence-free and long-term survival of prostate cancer patients

**DOI:** 10.1038/sj.bjc.6690806

**Published:** 1999-11

**Authors:** M Kuczyk, S Machtens, K Hradil, J Schubach, W Christian, R Knüchel, J Hartmann, C Bokemeyer, U Jonas, J Serth

**Affiliations:** Department of Urology, Hannover University Medical School, Carl Neubergstr. 1, Hannover 30625, Germany; Department of Hematology/Oncology, Eberhard-Karls-University, Tübingen, Germany; Department of Pathology, University of Regensburg, Regensburg, Germany

**Keywords:** prostate cancer, prognosis, *p27^Kip1^* gene

## Abstract

The *p27^Kip1^* gene has been identified as inductor of cell cycle arrest at the G1 checkpoint to prevent entry of somatic cells into the S phase of the cell cycle when substantial DNA damage has occurred. It has been suggested that decreased expression of the p27^Kip1^ protein may contribute to the development of human malignancies due to loss of critical antiproliferative mechanisms. In the present study, 95 specimens (T1–T4) from 95 randomly selected patients undergoing radical prostatectomy at the Urological Department of Hannover University (82 patients) as well as in the Josef Hospital Regensburg (13 patients) between 1981 and 1992 for whom tissue blocks for immunohistochemical investigation were available, were investigated for different biological and clinical characteristics as possible predictors for recurrence-free and long-term survival: age, depth of tumour infiltration, histological grade, lymph node status, as well as decreased expression of the p27^Kip1^ protein. After a median follow-up up of 56 months (24–151 months), seven of 21 (33%) patients (Group 1) with loss of p27^Kip1^ protein expression or a relative amount of <10% of positively stained tumour cells developed recurrent disease in contrast to 17 of 74 (23%) patients (Group 2) with retained p27^Kip1^ protein expression (≥10% of positively stained tumour cells). The median recurrence-free survival was 14 months (5–40 months) for patients from Group 1 and 31 months (7–133 months) for Group 2 patients (*P* = 0.02). In multivariate analysis, loss of p27^Kip1^ protein expression was identified as the only independent prognostic parameter for recurrence-free survival. In contrast, neither the univariate nor the multivariate analysis showed a correlation between loss of p27^Kip1^ protein expression and the long-term survival of the patients. Prospective studies are urgently needed to confirm the independent prognostic value of decreased p27^Kip1^ protein expression together with overexpression of the p53 tumour suppressor protein in patients with localized prostate cancer. The availability of more refined prognostically important biological variables in addition to established prognostic factors like tumour stage or Gleason score might help decision making in patients at high risk for the development of local recurrence or systemic tumour progression. © 1999 Cancer Research Campaign


*British Journal of Cancer* (1999) **81**(6), 1052–1058
© 1999 Cancer Research Campaign
Article no. bjoc.1999.0806

### Predictive value of decreased p27$^{Kip1}$ protein expression for the recurrence-free and long-term survival of prostate cancer patients

**M Kuczyk[1], S Machtens[1], K Hradil[1], J Schubach[1], W Christian[1], R Knüchel[3], J Hartmann[2], C Bokemeyer[2], U Jonas[1] and J Serth[1]**

[1]Department of Urology, Hannover University Medical School, Carl Neubergstr. 1, 30625 Hannover, Germany; [2]Department of Hematology/Oncology, Eberhard-Karls-University, Tübingen, Germany; [3]Department of Pathology, University of Regensburg, Regensburg, Germany

**Summary** The *p27$^{Kip1}$* gene has been identified as inductor of cell cycle arrest at the G1 checkpoint to prevent entry of somatic cells into the S phase of the cell cycle when substantial DNA damage has occurred. It has been suggested that decreased expression of the p27$^{Kip1}$ protein may contribute to the development of human malignancies due to loss of critical antiproliferative mechanisms. In the present study, 95 specimens (T1–T4) from 95 randomly selected patients undergoing radical prostatectomy at the Urological Department of Hannover University (82 patients) as well as in the Josef Hospital Regensburg (13 patients) between 1981 and 1992 for whom tissue blocks for immunohistochemical investigation were available, were investigated for different biological and clinical characteristics as possible predictors for recurrence-free and long-term survival: age, depth of tumour infiltration, histological grade, lymph node status, as well as decreased expression of the p27$^{Kip1}$ protein. After a median follow-up up of 56 months (24–151 months), seven of 21 (33%) patients (Group 1) with loss of p27$^{Kip1}$ protein expression or a relative amount of <10% of positively stained tumour cells developed recurrent disease in contrast to 17 of 74 (23%) patients (Group 2) with retained p27$^{Kip1}$ protein expression (≥10% of positively stained tumour cells). The median recurrence-free survival was 14 months (5–40 months) for patients from Group 1 and 31 months (7–133 months) for Group 2 patients ($P$ = 0.02). In multivariate analysis, loss of p27$^{Kip1}$ protein expression was identified as the only independent prognostic parameter for recurrence-free survival. In contrast, neither the univariate nor the multivariate analysis showed a correlation between loss of p27$^{Kip1}$ protein expression and the long-term survival of the patients. Prospective studies are urgently needed to confirm the independent prognostic value of decreased p27$^{Kip1}$ protein expression together with overexpression of the p53 tumour suppressor protein in patients with localized prostate cancer. The availability of more refined prognostically important biological variables in addition to established prognostic factors like tumour stage or Gleason score might help decision making in patients at high risk for the development of local recurrence or systemic tumour progression.
© 1999 Cancer Research Campaign

**Keywords:** prostate cancer; prognosis; *p27$^{Kip1}$* gene

Adenocarcinoma of the prostate is one of the most frequently diagnosed malignancies in Western countries and, following lung cancer, it has become one of the most common causes of death from cancer within the male population (Parker et al, 1996). While some patients die soon after diagnosis, other patients with 'latent' tumours will never suffer from any symptoms during their lifetime. This observation indicates the highly variable biological potential of prostate cancer and demonstrates the need for prognostic factors to determine the clinical prognosis of the individual patient and to guide currently available treatment options to a more aggressive (radical prostatectomy) or conservative (surveillance) approach (George, 1988; Adolfsson et al, 1992; Johanssen et al, 1992; Ackerman et al, 1993; Stamey et al, 1993). Recent investigations have tried to gain an improved understanding of tumour cell biology in order to determine the usefulness of biomarkers such as cell cycle associated proteins like p27$^{Kip1}$ or p21$^{WAF/Cip1}$ as prognostic factors in addition to classic pathological parameters (Gleason grade, T-stage, extracapsular growth, positive margins following radical prostatectomy) (Stamey et al, 1993).

*Received 10 September 1998*
*Revised 15 February 1999*
*Accepted 22 April 1999*

*Correspondence to:* M Kuczyk

Malignant transformation of normal somatic cells results from a multistep process that includes the loss of an intact cell cycle control, enhanced cellular proliferation, a lack of the induction of apoptosis and a decreased DNA repair. A group of specific proteins, the cyclins and cyclin-dependent kinases (cdks), are necessary for the induction of DNA replication following the formation of cyclin–cdk complexes that are activated by phosphorylation. The improved understanding of the mammalian cell cycle has shown the loss of cell cycle control being involved in the development and progression of malignancy (Nasmyth et al, 1996).

The cell cycle is controlled at two checkpoints, one at the G1/S and another at the G2/M transition. The function of these checkpoints is to avoid DNA replication or entry into mitosis when substantial DNA damage has occurred (Elledge, 1996; Kawasaki et al, 1996; Lee et al, 1997). Presently, the best understood checkpoint is located at the threshold from G1 to S phase transition.

To date, seven genes, namely *p15$^{mts2/ink4B}$*, *p16$^{cdkn2/ink4A}$*, *p18*, *p19*, *p21$^{WAF1/CIP1}$*, *p27$^{Kip1}$* and *p57*, have been identified as inductors of cell cycle arrest at the G1 checkpoint by a negative regulatory influence on cyclins and cdks (Kawamata et al, 1996). Recently, a heat stable 27 kDa protein, the transcript of the *p27^Kip1* gene that has been mapped on chromosome 12p12.3 (Martin et al, 1995), has been suggested to substantially participate in cell cycle control by preventing entry of somatic cells into the S phase of the cell cycle (Craig et al, 1997). $p27^{Kip1}$, first identified in extracts of tumour growth factor β (TGF-β)-treated cells as an inhibitor of cyclin E-cdk2 (Polyak et al, 1994*a*), binds to and inhibits the activity of multiple cyclin–cdk complexes, including cyclin E–cdk2, cyclin D–cdk4 and cyclin A–cdk2 (Polyak et al, 1994*a*, 1994*b*; Bullrich et al, 1995).

For patients with breast, small-cell lung and colorectal cancer decreased expression of the $p27^{Kip1}$ protein has been identified as a predictor of a decreased long-term survival (Esposito et al, 1997; Fredersdorf et al, 1997; Porter et al, 1997; Wu et al, 1997). In an initial investigation, loss of physiological $p27^{Kip1}$ protein expression has been discussed as a prognostically important biological variable only for the recurrence-free survival of patients undergoing radical prostatectomy for the treatment of clinically localized prostate cancer. In contrast, for 95 patients with tumours exclusively classified as stage C during the histopathological examination, Cote et al (1998) correlated altered expression of the $p27^{Kip1}$ protein both with the recurrence-free and overall survival following radical prostatectomy. The purpose of the present study was to further evaluate the involvement of $p27^{Kip1}$ in the development and progression of prostate cancer and to determine its possible value as a predictor of the recurrence-free and overall survival of 95 patients surgically treated for clinically localized tumours of different stage (T2–T4) and histological grading.

### PATIENTS AND METHODS

### Patients

Ninety-five consecutive patients undergoing radical prostatectomy at the Urological Department of Hannover University Medical School between 1981 and 1994 for the treatment of clinically localized prostate cancer for whom fresh-frozen or paraffin-embedded tissue sections for immunohistochemical analysis were available, were included in the present investigation. Prior to radical prostatectomy, the presence of distant metastases was excluded by abdominal computerized tomography (CT) scans, X-rays of the lungs and bone scans. The median age of the patients was 63 years (45–78 years). Following pelvic lymph node dissection and radical prostatectomy, tumour specimens were reviewed by one pathologist and classified as T2 (49 patients, 45%), T3 (43 patients, 45%) and T4 (three patients, 4%) according to the TNM system (Hermanek et al, 1993). Six of 49 patients with organ-confined tumours (T2) underwent radical prostatectomy due to the diagnosis of an incidental carcinoma (T1) during a previously performed transurethral resection for the treatment of benign prostatic hyperplasia (BPH). The tumours were histologically graded as G1 (nine tumours, 9%), G2 (58 tumours, 61%) and G3 (28 tumours, 30%).

The final histopathological examination of the dissected regional lymph nodes, in all patients classified as tumour-free during intra-operatively performed histopathological examination, revealed regional lymph node metastases in eight cases (N1, six patients; N2, two patients). All patients were followed after prostatectomy by transrectal ultrasound (after availability of this diagnostic approach), bone scans and determination of the serum prostate-specific antigen (PSA) (Hybritech, Germany) and prostate acid phosphatase (PAP) (in patients treated before availability of a serum PSA assay) levels every 6 months. The median follow-up after radical prostatectomy was 56 months (24–151 months).

In case of a suspicious digito-rectal examination (DER) or rising PSA levels of PSA or PAP during follow-up, patients received a CT scan of the abdomen, X-ray of the lungs and a bone scan. In case of a rising serum PSA level on two consecutive tests ($\geq$ 0.4 ng $ml^{-1}$) without the detection of lymph node or distant metastases needle biopsies were obtained from the vesicourethral anastomosis to diagnose or exclude local recurrence. In cases of a systemic tumour progression (20 patients) patients were treated with androgen ablation (CAB) either by bilateral orchiectomy or by the administration of a luteinizing hormone-releasing hormone (LHRH) analogue in combination with flutamide.

### Immunohistochemistry

Formalin-fixed and paraffin-embedded (21 tumours) as well as fresh-frozen tissue sections (74 tumours) were investigated for expression of the $p27^{Kip1}$ and $p21^{WAF/Cip}$ proteins by an immunohistochemical approach. As an internal negative control for the staining procedure, each tumour in the study was incubated with non-immune mouse IgG instead of the primary antibody, followed by the identical procedure for the application of the secondary antibodies. Quiescent Balb/c 3T3 cells, induced rat embryo fibroblasts as well as tissue samples obtained from ten patients undergoing surgery for the treatment of BPH served as biological positive controls respectively. In the latter tissue specimens a relative amount of > 70% of epithelial cells exhibited a positive immunohistochemical staining reaction for the $p27^{Kip1}$ protein (Figure 1).

During a preliminary study, fresh-frozen as well as formalin-fixed serial cuttings obtained from ten prostate cancer specimens

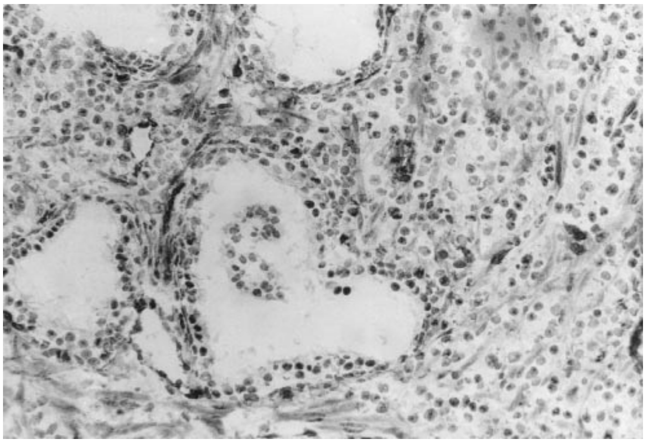

**Figure 1** Immunohistochemically detected expression of the $p27^{Kip1}$ protein in a tissue sample obtained from a benign prostatic hyperplasia specimen with a relative amount of > 70% of epithelial cells exhibiting a positive staining reaction (magnification 240-fold, ABC method)

© 1999 Cancer Research Campaign were immunohistochemically investigated for the p27$^{Kip1}$ protein to determine the influence of the kind of tissue fixation on the immunohistochemical staining reaction. Although the immunohistochemical staining reaction observed in paraffin-embedded tissue sections following incubation in citrate buffer for antigen retrieval revealed a slightly higher intensity when compared with the frozen-frozen tissue specimens, the staining pattern regarding the amount of positively stained tumour cells was absolutely identical, hereby indicating the applicability of the anti-p27$^{Kip1}$ antibody in fresh-frozen as well as in paraffin-embedded tissue.

Following dewaxing, paraffin-embedded sections as well as fresh-frozen slides were cut serially at 4 µm resp. 8-µm thickness and stained for the p27$^{Kip1}$ protein. Paraffin sections were picked up on 3-aminopropyltri-ethoxysilan (APES)-coated slides, dried for 1 day at room temperature and for an additional 3–4 h at 40°C. For antigen retrieval, sections were incubated with 0.1 M citrate buffer (pH 6) for 5–6 h at 70°C. Endogenous peroxidase activity was blocked by incubation for 30 min at room temperature in 3% hydrogen peroxidase diluted in phosphate-buffered saline (PBS) (0.5 M, pH 7.4). After rinsing in PBS–0.1% Tween-20 the tumour-bearing slides were incubated with normal human serum at a dilution of 1:100 in PBS for 30 min to prevent non-specific binding of the first antibody. Then the specific monoclonal primary antibody for the detection of the p27$^{Kip1}$ protein (Clone G173–524, $IgG_1$; Pharmingen, San Diego, CA, USA) was added. As indicated by the manufacturer, the anti-p27$^{Kip1}$ antibody is generated by immunizing mice with a full-length recombinant mouse His-fusion protein. The anti-p27$^{Kip1}$ antibody was applied at a dilution of 1:50 in PBS at room temperature for 1 h in a moist chamber respectively. After rinsing with PBS–0.1% Tween-20 for 10 min a standard streptavidin–biotin complex (Vectastain, Burlingame, CA, USA) method was applied according to the instructions of the manufacturer.

**Table 1** Prostate cancers investigated for the correlation between decreased expression of the p27$^{Kip}$ protein and the recurrence-free as well as long-term survival following radical prostatectomy

| Patients' characteristics | Group 1 (< 10% positivity) *n* = 21 (22%) | Group 2 (≥ 10% positivity) *n* = 74 (78%) |
|---|---|---|
| Age (years) | 62 (48–71) | 61 (45–78 years) |
| Tumour stage (T) | | |
| Pat. *n* (%) | | |
| pT1 | 1 (4) | 5 (6) |
| pT2 | 10 (48) | 33 (45) |
| pT3 | 10 (48) | 33 (45) |
| pT4 | 0 | 3 (4) |
| Histological grade (G) | | |
| Pat. *n* (%) | | |
| G1 | 1 (5) | 8 (11) |
| G2 | 13 (62) | 45 (61) |
| G3 | 7 (33) | 21 (28) |
| Follow-up (months) | 49 (24–138) | 59 (24–151) |
| Tumour recurrence | | |
| Pat. *n* (%) | 7 (33) | 17 (23) |
| Tumour-dependent | | |
| death | 4 (19) | 16 (22) |
| Pat. *n* (%) | | |
| Recurrence-free survival (months) | 14 (5–40) | 31 months (7–133 months) |
| Long-term survival | 33 (31–81) | 59 months (4–151 (months) |

Detailed characterization of patients from Group 1 (< 10% positivity) and Group 2 (≥ 10% positivity). Patients from Group 1 and 2 were comparable regarding other characteristics of possible prognostic value ($\chi^2$).

### Classification of immunohistochemistry

Depending on the percentage of nuclei exhibiting a positive immunohistochemical staining reaction for the p27$^{Kip1}$ protein, the tumours were classified into two groups: (1) negative reaction or < 10% positivity regarding the relative amount of positively stained tumour cells, (2) ≥ 10% positivity. This cut-off value was based on the previously reported experience regarding the most suitable classification of immunohistochemistry in breast and colorectal cancers. For the classification of the immunohistochemical staining reaction, in five microscopic fields (magnification 240-fold) per tissue slide, 400–500 tumour cells were counted irrespective of the result of the immunohistochemical staining reaction. For analytical purposes, the highest category obtained in each patient was considered. The immunohistochemical staining reaction was uniformly identified within the cell nuclei. Five separate slides per patient were reviewed and classified by two independent investigators. For the classification of the immunohistochemical staining reaction, the percentage of positively stained tumour cells observed in each of these five tissue slides was estimated in correlation to the total number of tumour cells identified.

### Statistical calculations

For p27$^{Kip1}$ immunohistochemistry, patients were divided into groups revealing </≥ 10, </≥ 40 and </≥ 60% positivity and for each of these cut-off levels, the predictive value of a decreased p27$^{Kip1}$ protein expression regarding the amount of positively stained tumour cells was calculated for the recurrence-free as well as the long-term survival of patients. Spearman correlation coefficients were determined to correlate p27$^{Kip1}$ protein expression with further patients' and tumour characteristics (e.g. histological grading, tumour stage, age and preoperative serum PSA level). Univariate analysis using a log-rank test was employed for each possible prognostic factor alone to determine its prognostic significance for recurrence-free and overall survival of the patients. Tumour-free survival was calculated from the time of radical prostatectomy to either relapse in the form of a rising PSA level (≥ 0.4 ng ml$^{-1}$ on two consecutive tests determined with an interval of at least 50 days) or the diagnosis of local recurrence (confirmed by the obtainment of a needle biopsy from the vesicourethral anastomosis) or metastatic lesions. Overall survival was calculated according to the Kaplan–Meier method from the time of radical prostatectomy to either death or date of last follow-up. Finally, multivariate Cox proportional hazards model was used to determine whether any of the factors tested, age, tumour stage, histological grade or percentage of immunohistochemical positivity for the p27$^{Kip1}$ protein could be identified as independent prognostic factors for the recurrence-free and the long-term survival of the patients. The comparability of patients exhibiting </≥ 10% positivity for the p27$^{Kip1}$ protein regarding other patients' or tumour characteristics of possible prognostic value was determined using a $\chi^2$ test (Table 1).

 *© 1999 Cancer Research Campaign*

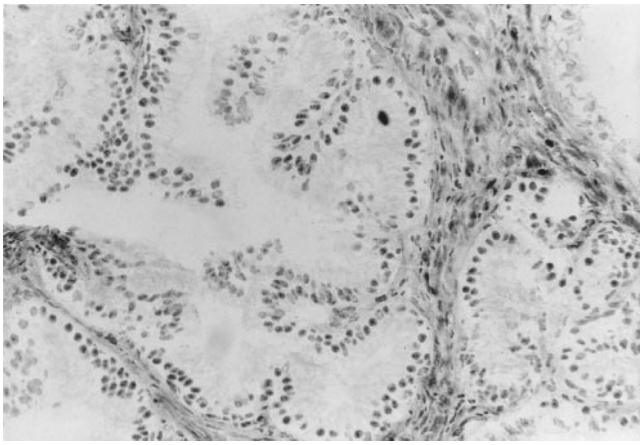

**Figure 2** Complete loss of p27Kip1 protein expression in a radical prostate cancer specimen (T3,G2) (ABC method, magnification 240-fold)

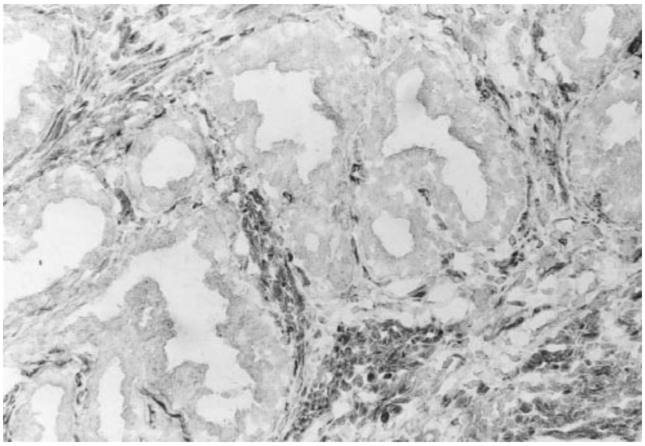

**Figure 3** Immunohistochemically detected staining pattern for the p27Kip1 protein in a radical prostate cancer specimen (T3,G2): retained expression of the p27Kip1 protein in > 10% of tumour cells (ABC method, magnification 240-fold)

### RESULTS

### Association of p27Kip1 protein expression with recurrence-free survival

Univariate analysis using the log-rank test was employed for each biological variable alone to determine its significance for recurrence-free survival. Out of 95 prostate cancer specimens investigated for p27Kip1 protein expression, 21 (22%) patients exhibited a completely negative (Figure 2) or a positive staining reaction in < 10% of tumour cells (Group 1) and 74 (78%) patients (Group 2) a retained immunohistochemical staining reaction in at least 10% of tumour cells (Figure 3). Nuclear reactivity for the p27Kip1 protein was seen in the luminal cells as well as in the basal cell layers of all BPH samples investigated.

Seven of 21 (33%) patients with a negative staining reaction or a retained p27Kip1 protein expression in < 10% of tumour cells (Group 1) developed tumour progression compared with 17 of 74 (23%) patients from Group 2 (≥ 10 positivity) (median follow-up was 49 months (24–138 months) for patients from Group 1 and 59 months (24–151 months) for Group 2 patients). The median recurrence-free survival following radical prostatectomy was 14 months (5–40 months) for Group 1 patients and 31 months (7–133 months) for patients from Group 2 ($P$ = 0.02; log-rank test) (Table 2, Figure 4). There were no imbalances for other investigated variables between the two groups (Table 1). A negative staining reaction as a predictor of recurrence-free survival did not achieve statistical significance at the other cut-off values (</≥ 40 and </≥ 60% positivity) calculated (data not shown).

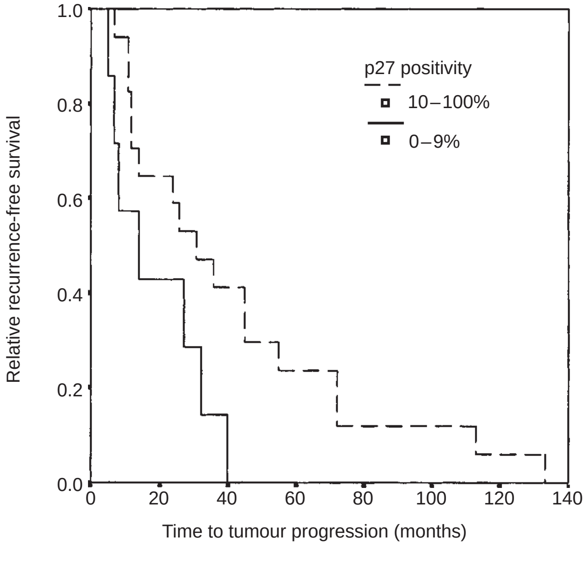

**Figure 4** Recurrence-free survival following radical prostatectomy, calculated according to the Kaplan–Meier method. Classification into Group 1 (< 10% positively stained tumour cells) and Group 2 (≥ 10% positivity): Group 2 patients had a significantly decreased recurrence-free survival following radical prostatectomy ($P$ = 0.02)

### Statistical analysis of further prognostic parameters

Univariate analysis demonstrated that tumour-free survival following radical prostatectomy was independent of age ($P$ = 0.12), histological grading ($P$ = 0.07), the presence of regional lymph node metastases ($P$ = 0.45), as well as the serum PSA level ($P$ = 0.23). Tumour stage ($P$ = 0.03) and, with a cut-off value of 10%, retained expression of the p27Kip1 protein was significantly correlated with the recurrence-free survival of the patients ($P$ = 0.02). In multivariate analysis, reactivity for the p27Kip1 protein ($P$ = 0.04) proved to be the only statistically relevant prognostic factor for the recurrence-free survival of the patients.

Spearman correlation coefficients were calculated to determine if p27Kip1 positivity could be correlated with further tumour or patient's characteristics. Immunohistochemically detected expression of the p27Kip1 protein was demonstrated as a variable independent from tumour stage ($P$ = 0.31), histological grading

© 1999 Cancer Research Campaign

**Table 2** Patients' and tumour characteristics evaluated as parameters of possible prognostic importance for the recurrence-free and overall survival of 95 patients undergoing radical prostatectomy for the treatment of clinically localized prostate cancer

| Factor investigated | Univariate analysis (log-rank test) | Multivariate analysis (Cox regression) |
|---|---|---|
| Recurrence-free survival following radical prostatectomy | | |
| Tumour stage | Yes ($P$ = 0.03) | No ($P$ = 0.09) |
| Histological grading | No ($P$ = 0.07) | No ($P$ = 0.32) |
| Lymph node status | No ($P$ = 0.45) | No ($P$ = 0.83) |
| Patients' age | No ($P$ = 0.12) | No ($P$ = 0.14) |
| IHC for $p27^{Kip1}$ (cut-off value 10%) | Yes ($P$ = 0.02) | Yes ($P$ = 0.04) |
| Overall survival following radical prostatectomy | | |
| Tumour stage | Yes ($P$ = 0.04) | No ($P$ = 0.31) |
| Histological grading | Nos ($P$ = 0.08) | No ($P$ = 0.23) |
| Lymph node status | No ($P$ = 0.18) | No ($P$ = 0.57) |
| Patient's age | No ($P$ = 0.23) | No ($P$ = 0.48) |
| IHC for $p27^{Kip1}$ (cut-off value 1%) | No ($P$ = 0.12) | No ($P$ = 0.19) |

IHC: immunohistochemistry.

($P$ = 0.37), patients age ($P$ = 0.34), the serum PSA level ($P$ = 0.64) and the lymph node status ($P$ = 0.62).

### Association of $p27^{Kip1}$ protein expression with overall survival

After a median follow-up of 56 months (24–151 months), 20 of 95 (21%) patients had died from tumour progression. During the follow-up period, four of 21 (19%) patients from Group 1 (negative reaction or < 10% positivity) died from tumour progression, in contrast to 16 of 74 (22%) patients from Group 2 (≥ 10% positivity for the $p27^{Kip1}$ protein). The calculated median survival times were 33 months (31–81 months) for patients from Group 1 and 59 months (4–151 months) for Group 2 patients. For one of the patients classified into Group 2 who died from tumour progression 4 months after surgery, the final histopathological examination of the intraoperatively dissected lymph nodes revealed regional lymph node metastases (N2). Although there was a tendency towards a decreased long-term survival for patients revealing loss of $p27^{Kip1}$ protein expression within the primary tumours, the difference between Group 1 and Group 2 patients was not statistically significant ($P$ = 0.12) (Table 2). Accordingly, the level of statistical significance for overall survival was not reached with all other cut-off values (</≥ 40 and </≥ 60%) for the amount of positively stained tumour cells (data not shown).

### Statistical analysis of further prognostic parameters

Univariate statistical analysis demonstrated that the time of survival following radical prostatectomy was independent of age ($P$ = 0.23), the diagnosis of regional lymph node metastases ($P$ = 0.34), the histological grading ($P$ = 0.08), the preoperatively determined serum PSA level ($P$ = 0.31) and positivity for the $p27^{Kip1}$ protein ($P$ = 0.12). Tumour stage was the only biological variable significantly correlated with the long-term survival of the patients ($P$ = 0.04). In a multivariate analysis, none of the aforementioned parameters was identified as an independent prognostic factor for overall survival.

### DISCUSSION

For the majority of human malignancies, previously reported investigations in several human tumours including prostate cancer failed to demonstrate any alteration at the *p27^Kip1* gene locus (Bullrich et al, 1995; Ferrando et al, 1996; Spirin et al, 1996). Therefore, reduced $p27^{Kip1}$ protein expression as meanwhile identified in a variety of human malignancies including colorectal carcinomas, breast cancer and non-small-cell lung cancer specimens (Fredersdorf et al, 1997; McGarvey and Malkowicz, 1997; Yasui et al, 1997), has been initially suggested to result from a post-translational regulatory influence of growth factors like TGFβ or cAMP (Kato et al, 1994; Ferrando et al, 1996; Spirin et al, 1996; Esposito et al, 1997; Yasui et al, 1997). Recently, it has been suggested that the lower or even undetectable protein level in tumorous tissue specimens when compared with normal somatic cells more likely results from an increased degradation of the $p27^{Kip1}$ protein than from an altered $p27^{Kip1}$ gene transcription or $p27^{Kip1}$ mRNA stability (Ponce-Castaneda, 1995).

Therefore, the investigation of cancerous tissue specimens for $p27^{Kip1}$ alterations on the protein level by immunohistochemistry, for example, appears as the most suitable analytical approach to clarify the possible involvement of $p27^{Kip1}$ in the development and progression of human malignancies and to further evaluate the role as a biological variable of possible prognostic importance (Cote et al, 1998).

Whereas for non-small-cell lung cancer, colorectal and breast cancers (Esposito et al, 1997; Porter et al, 1997), decreased $p27^{Kip1}$ protein expression was correlated with a shorter overall survival of the patients, Fredersdorf et al (1997) also reported contradictory findings for a subset of highly proliferative breast cancer cell lines exhibiting high level $p27^{Kip1}$ expression. An initial study in bladder cancer, investigating the expression of $p27^{Kip1}$ mRNA in 14 superficial and 14 muscle invasive tumour specimens, reported a decreased level of the $p27^{Kip1}$ transcript in invasive compared with superficial lesions (McGarvey et al, 1997).

To date, the prognostic value of a decreased $p27^{Kip1}$ protein expression for patients undergoing radical prostatectomy for the treatment of clinically localized prostate cancer has not yet been well determined. Initially, Yang et al (1998) identified absent or low $p27^{Kip1}$ protein expression as an adverse prognostic parameter for patients with clinically organ-confined prostate cancer (Yang et al, 1998). However, loss of $p27^{Kip1}$ protein expression was not identified as a predictor of long-term survival following radical prostatectomy (Yang et al, 1998). Recently, Cote et al (1998) investigated the prognostic value of decreased $p27^{Kip1}$ protein expression for the recurrence-free and overall survival of 96 stage C prostate cancer patients (median follow-up: 9.6 years) undergoing radical prostatectomy. For a cut-off value of 10%, loss of $p27^{Kip1}$ protein expression was inversely correlated with the Gleason score and clearly identified as an independent prognostic parameter both for the recurrence-free and long-term survival of the patients.

In the present investigation the median recurrence-free survival following radical prostatectomy was 14 months (5–40 months) and 31 months (7–133 months) for patients without and with retained

 *© 1999 Cancer Research Campaign*

expression (≥ 10% positivity) of the p27$^{Kip1}$ protein. This difference proved to be statistically significant ($P$ = 0.02). Whereas in univariate statistical analysis tumour stage in combination with altered p27$^{Kip1}$ protein expression was identified as predictor of an early tumour recurrence following radical prostatectomy, during multivariate analysis loss of p27$^{Kip1}$ protein expression was identified as the only independent prognostic predictor of recurrence-free survival.

However, in contrast to the results reported by Cote et al (1998) and in accordance with the observation by Yang et al (1998), in our study decreased expression of the p27$^{Kip1}$ protein was not correlated to the long-term survival of the patients. This observation might be explained with the high frequency of low-stage tumours (≤ T2) (52%) included in our study, whereas the investigation by Cote et al (1998) exclusively evaluated the prognostic importance of p27$^{Kip1}$ in locally advanced (Stage C) prostate cancer specimens. Additionally, Cote et al (1998) do not give any information on the kind of adjuvant therapy applied in case of local recurrence or systemic tumour progression. Due to the role of the p27$^{Kip1}$ protein during cell cycle regulation its ability to predict tumour responsiveness regarding hormone ablation or adjuvant radiotherapy appears at least questionable. Therefore, according to our study and with regard to the results reported by Yang et al (1998), the observation of decreased p27$^{Kip1}$ protein expression might rather help identifying patients at high risk for tumour recurrence or systemic progression who urgently need early adjuvant therapy.

For clinically localized prostate cancer recent studies strongly indicate that the determination of p53 inactivation allows the identification of a highly aggressive subgroup of prostatic tumours with decreased recurrence-free and long-term survival following radical prostatectomy (Kuczyk et al, 1998). The outcome of the present study seems to indicate that in addition to p53 and already established prognostic parameters like Gleason score or tumour stage, the detection of altered p27$^{Kip1}$ expression might help to establish a therapeutic regimen adjusted to the aggressiveness of the individual tumour, although this result will have to be confirmed during further prospective investigations including a range of prognostically important biological variables like p27$^{Kip1}$ or p53. Therefore, further insight in the role of cell cycle associated proteins for the development and progression of prostate cancer might contribute to the establishment of effective approaches aiming at the reconstitution of functionally altered cell cycle regulatory mechanisms.

### ACKNOWLEDGEMENTS

JS was supported by Deutsche Forschungsgemeinschaft (DFG).

### REFERENCES

Ackerman DA, Barry JM, Wicklund RA, Olson N and Lowe BA (1993) Analysis of high risk factors associated with prostate cancer extension to the surgical margin and pelvic node metastasis at radical prostatectomy. *J Urol* **150**: 1845–1850

Adolfsson J, Carstensen T and Lowhagen T (1992) Deferred treatment in localised prostate cancer. *Br J Urol* **69**: 183–187

Bullrich FF, McLachlan TK, Sang N, Druck T, Veronese ML, Allen SL, Chiorazzi N, Koff A, Heubner K, Croce CM and Giordano A (1995) Chromosomal mapping of members of the cdk2 family of protein kinase, cdk3, cdk6, PISSLRE, and PITALRE, and a cdk inhibitor, p27$^{Kip1}$, to regions involved in human cancer. *Cancer Res* **55**: 1199–1205

Cote R, Shi Y, Groshen S, Feng A-C, Cordon-Cardo C, Skinner D and Lieskovsky G (1998) Association of p27$^{Kip1}$ levels with recurrence and survival in patients with stage C prostate carcinoma. *J Natl Cancer Inst* **90**: 916–920

Craig C, Wersto R, Kim M, Ohri E, Li Z, Katayose D, Lee SJ, Trepel J, Cowan K and Seth P (1997) A recombinant adenovirus expressing p27$^{Kip1}$ induces cell cycle arrest and loss of cyclin–cdk activity in human breast cancer cells. *Oncogene* **14**: 2283–2289

Elledge SJ (1996) Cell cycle checkpoints: preventing an identity crisis. *Science* **274**: 1664–1672

Esposito V, Baldi A, De Luca A, Groger AM, Loda M, Giordano GG, Caputi M, Baldi F, Pagano M and Giordano A (1997) Prognostic role of the cyclin-dependent kinase inhibitor p27 in non-small cell lung cancer. *Cancer Res* **57**: 3381–3385

Ferrando AA, Balbin M, Pendas AM, Vizoso F, Velasco G and Lopez-Otin C (1996) Mutational analysis of the human cyclin-dependent kinase inhibitor p27Kip1 in primary breast carcinomas. *Hum Genet* **97**: 91–94

Fredersdorf S, Burns J, Milne AM, Packham G, Falls L, Gillett CE, Royds JA, Peston D, Hall PA, Hanby AM, Barnes DM, Shousha S, O' Hare MJ and Lu X (1997) High level expression of p27(kip1) and cyclin D1 in some human breast cancer cells: inverse correlation between the expression of p27(kip1) and degree of malignancy in human breast and colorectal cancers. *Proc Natl Acad Sci USA* **94**: 6380–6385

George NJR (1988) Natural history of localised prostate cancer managed by conservative therapy alone. *Lancet i*: 494–497

Hermanek P and Sobin LH (eds.) (1992) *UICC TNM Classification of Malignant Tumours*, 4th edn, 2nd revision. Springer, Berlin.

Johansson JE, Adami HO, Andersson SO, Bergström R, Holmberg L and Krusemo UB (1992) High 10-year survival rate in patients with early untreated prostate cancer. *JAMA* **267**: 2191–2196

Kato J, Matsuoka M, Polyak K, Massague J and Sherr CJ (1994) Cyclic AMP-induced G1 phase arrest mediated by an inhibitor (p27$^{kip1}$) of cyclin-dependent kinase 4 activation. *Cell* **79**: 487–496

Kawamata N, Seriu T, Koeffler HP and Bartram CR (1996) Molecular analysis of the cyclin-dependent kinase inhibitor family: p16 (CDKN2/MTS1/INK4A), p18 (INK4C) and p27 (Kip1) genes in neuroblastomas. *Cancer* **77**: 570–575

Kawasaki T, Tomita Y, Bilim V, Takeda M, Takahashi K and Kumanishi T (1996) Abrogation of apoptosis induced by DNA-damaging agents in human bladder-cancer cell lines with p21/WAF1/CIP1 and/or p53 gene alterations. *Int J Cancer* **68**: 501–505

Kuczyk MA, Serth J, Bokemeyer C, Machtens S, Minssen A, Bathke W, Hartmann J and Jonas U (1998) The prognostic value of p53 for the long-term and recurrence-free survival following radical prostatectomy. *Eur J Cancer* **34**: 679–686

Lee CCR, Yamamoto S, Wanibuchi H, Wada S, Sugimura K, Kishimoto T and Fukushima S (1997) Cyclin D1 overexpression in rat two-stage bladder carcinogenesis and its relationship with oncogenes, tumor suppressor genes, and cell proliferation. *Cancer Res* **57**: 4765–4776

Martin E, Cacheux V, Cave H, Lapierre JM, Le Paslier D and Grandchamp B (1995) Localization of the CDKN4/p27(Kip1) gene to human chromosome 12p12.3. *Hum Genet* **96**: 668–670

McGarvey TW and Malkowicz SB (1996) The role of the cell cycle in genitourinary carcinoma. *World J Urol* **14**: 310–317

Nasmyth K (1996) Viewpoint: putting the cell cycle in order. *Science* **274**: 1643–1645

Parker SI, Tong T, Bolden S and Wingo PA (1996) Cancer Statistics 1996. *CA Cancer J Clin* **46**: 5–27

Polyak K, Kato J, Solomon MJ, Sherr CJ, Massague J, Roberts JM and Koff A (1994*a*) p27$^{Kip1}$ a cyclin–cdk inhibitor, links transforming growth factor-β and contact inhibition to cell cycle arrest. *Genes Dev* **8**: 9–22

Polyak K, Lee M-H, Erdjument-Bromage H, Koff A, Roberts JM, Tempst P and Massague J (1994*b*) Cloning of p27(Kip1), a cyclin-dependent kinase inhibitor and a potential mediator of extracellular antimitogenic signals. *Cell* **78**: 59–66

Ponce-Castaneda MV, Lee MH, Latres E, Polyak K, Lacombe L and Montgomery K (1995) P27 Kip1: chromosomal mapping to 12p12–12p13.1 and absence of mutations in human tumors. *Cancer Res* **55**: 1211–1214

Porter PL, Malone KE, Heagerty PJ, Alexander GM, Gatti LA, Firpo EJ, Daling JR and Roberts JM (1997) Expression of cell-cycle regulators p27Kip1 and cyclin E, alone and in combination, correlate with survival in young breast cancer patients. *Nat Med* **3**: 222–225

Spirin KS, Simpson JF, Takeuchi S, Kawamata N, Miller CW and Koeffler HP (1996) p27/Kip1 mutation found in breast cancer. *Cancer Res* **56**: 2400–2404

Stamey TA, Freiha FS, McNeal JE, Redwine EA, Whitemore AS and Schmid HP (1993) Localized prostate cancer: relationship of tumor volume to clinical significance for the treatment of prostate cancer. *Cancer* **71**: 933–938

© 1999 Cancer Research Campaign 

Wu G, Fan RS, Li W, Ko TC and Brattain MG (1997) Modulation of cell cycle control by vitamin D3 and its analogue, EB 1089, in human breast cancer cells. *Oncogene* **15**: 1555–1563

Yang RM, Naitoh J, Murphy M, Wang HJ, Phillipson J, De Kernion JB, Loda M and Reiter RE (1998) Low p27 expression predicts poor disease-free survival in patients with prostate cancer. *J Urol* **159**: 941–945

Yasui W, Kudo Y, Semba S, Yokozaki H and Tahara E (1997) Reduced expression of cyclin-dependent kinase inhibitor p27Kip1 is associated with advanced stage and invasiveness of gastric carcinomas. *Jpn J Cancer Res* **88**: 625–629

*© 1999 Cancer Research Campaign*
